# Teledentistry: A Comprehensive Review and Its Application in Pediatric Dental Care

**DOI:** 10.7759/cureus.52685

**Published:** 2024-01-21

**Authors:** Harikishan Kanani, Monika Khubchandani, Suwarna Dangore-Khasbage, Ruchika Pandey

**Affiliations:** 1 Pediatric Dentistry, Sharad Pawar Dental College and Hospital, Datta Meghe Institute of Higher Education and Research, Wardha, IND; 2 Oral Medicine and Radiology, Sharad Pawar Dental College and Hospital, Datta Meghe Institute of Higher Education and Research, Wardha, IND; 3 Orthodontics and Dentofacial Orthopedics, Sharad Pawar Dental College and Hospital, Datta Meghe Institute of Higher Education and Research, Wardha, IND

**Keywords:** pediatrics and preventive dentistry, urban and rural community, communication dentistry, telemedicine, teledentistry

## Abstract

In recent years, dental technology has experienced remarkable advancements, mirroring the evolution of communication and information technologies. The advent of information technology has paved the way for a new frontier in healthcare known as teledentistry. This innovative approach has revolutionized the delivery of dental care across geographical distances, enhancing accessibility and communication in the realm of oral health.

This article aims to highlight the various methodologies of teledentistry, discuss its benefits for both patients and dentists, and emphasize its potential to overcome geographical barriers, enhance access to dental care, and promote oral health equity, especially among children.

There are three primary teledentistry methods: real-time consultation, the store-and-forward method, and the remote monitoring method. Real-time consultation facilitates immediate interaction between dentists and patients through video conferencing, enabling the sharing of data and medical history. The store-and-forward method involves the collection and transmission of essential diagnostic materials, enhancing diagnostic accuracy. Remote monitoring allows continuous patient evaluation from a distance, improving healthcare delivery and patient safety. Teledentistry’s impact on pediatric and preventive dentistry is substantial. It offers remote counseling, diagnosis, and monitoring for children, especially in areas with limited access to dental care. Additionally, mobile gaming apps play a role in behavioral management and reducing dental anxiety among children. Both patients and dentists benefit from teledentistry. Patients gain access to timely consultations, reducing the need for immediate in-person visits. Dentists can efficiently evaluate and monitor patients, collaborate with specialists, and provide expert advice, leading to improved healthcare delivery.

Teledentistry is transforming dental care by overcoming geographical barriers, improving access, and enhancing communication. While facing challenges, its potential to revolutionize healthcare delivery and promote oral health equity is undeniable. With ongoing advancements and strategic measures, teledentistry is poised for a promising future in healthcare.

## Introduction and background

Dental technology has undergone a lot of technological advancement in recent years. Terms for remote healthcare services, telehealth, and telemedicine, for example, have changed through time, much like communication technologies and electronic information applications. With the introduction of information technology and, more crucially, its widespread use, a relatively new area has formed. They have their origins in telemedicine and bring up a new vision for dental innovation. An innovative practice model called teledentistry has great promise for the future of clinical care and the provision of public health services [1,2].

The term "teledentistry" was initially coined in 1997 and was introduced by Cook as "the utilization of video conferencing technologies for remote diagnosis and treatment guidance." Furthermore, it encompasses the domain of telemedicine specific to dentistry, encompassing comprehensive networking, digital information sharing, and remote consultations, as well as the process of evaluation and analysis [3]. This innovative approach has revolutionized how dental care is administered over geographical distances, facilitating enhanced accessibility and communication in the realm of oral health [4]. Teledentistry became an essential tool for delivering oral healthcare during the COVID-19 pandemic. It provided online consultations, assessments of dental problems, and patient assistance, avoiding in-person visits to dental clinics and reducing the risk of virus transmission. Teledentistry proved to be of great use during this period, as it not only provided remote consultations but also helped in the screening and triage of patients. It paved the way to help educate the patients regarding ways to prevent infection. Documentation of the patient's prescribing medications, and recall care was also made easy with this innovation. The most affordable, efficient, and specialized method for reaching economically underserved and inaccessible regions of a community is teledentistry. It could do away with the inequities in oral healthcare, advancing equity [5].

Teledentistry is built on the Internet. This subspecialist branch also benefits from the rapidity, economy, documented consultation, and simultaneous communication of multiple participants. There are several drawbacks, including a lack of adequate training, a demand for quick responses, misunderstandings caused by language barriers, and privacy concerns, which restrict its usage for the successful delivery of healthcare [6]. It aims to highlight the various methodologies of teledentistry, discuss its benefits for both patients and dentists, and emphasize its potential to overcome geographical barriers, enhance access to dental care, and promote oral health equity, especially among children.

## Review

Teledentistry methodologies

**Figure 1:**
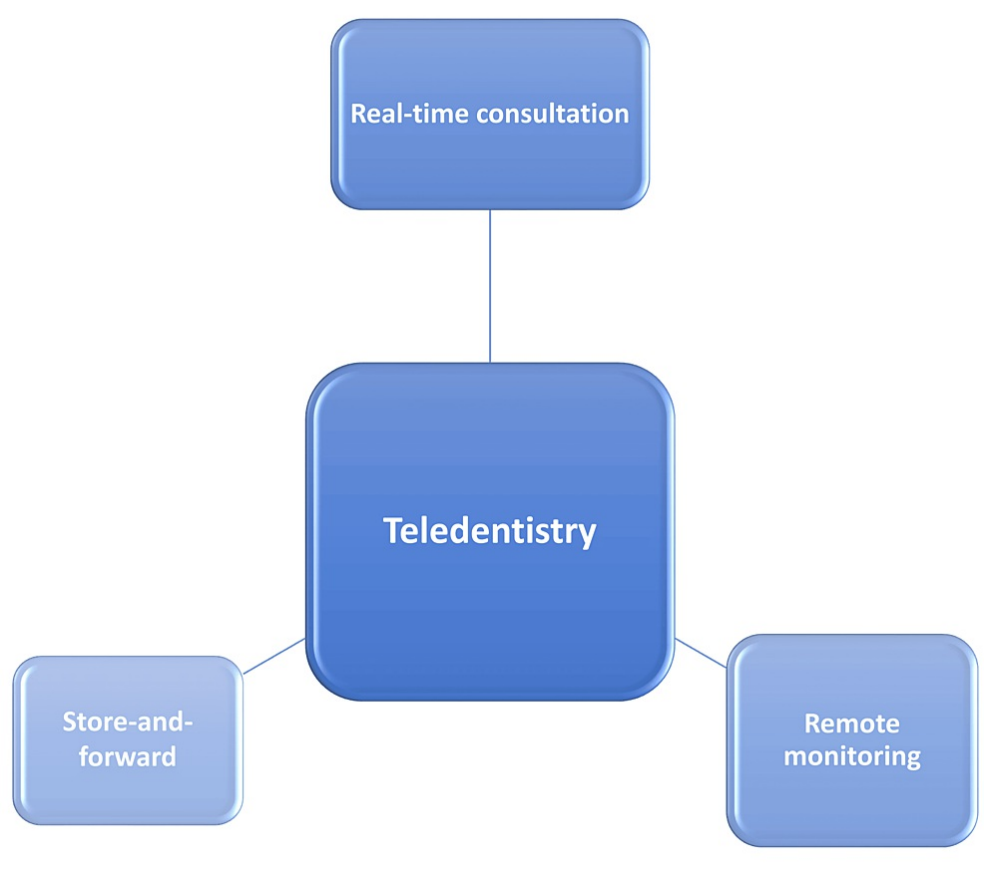
Methods of teledentistry

A teleconsultation can be conducted in three ways: real-time consultation, store-and-forward method, and remote monitoring method (Figure 1).

*Real-Time Consultation*

Real-time consultation comprises video conferencing between a dentist and a patient. Real-time consulting involves the simultaneous sharing of data, reports, and medical history. Additionally, it makes communication with a dentist or other specialist quicker and helps to share information. A person on an overseas or distant site can obtain real-time images or sound from the origination site using two-way interactive technology [7].

Store-and-Forward Method

The second approach is the "store-and-forward method," in which a treating consultant gets data from one area, saves it, and then transmits it to another consultant and plans the treatment. Essential diagnostic materials, such as X-rays, photographs, and scanned images, are meticulously gathered and presented to the consultant following a thorough examination. These visual aids play a crucial role in facilitating accurate assessment and decision-making. The information can be securely stored within the system for future reference, ensuring a comprehensive record of the patient's dental health journey. This integration of advanced imaging technology into teledentistry enhances the precision and effectiveness of consultations in the realm of pedodontics, contributing to optimal care for young patients. Before referring or monitoring a patient, utilize this strategy to contact an expert or team in another region, city, or country. After previous authorization, the data may be used to alert colleagues. Many websites also offer a style of asynchronous consultation where users can enter serology or pathology reports to retrieve a potential diagnosis [8].

Remote Monitoring Method

The third technique is called the "remote monitoring method," where patients are continuously examined from a hospital or at home. For the sake of healthcare and administrative assistance, medical data and other health information are transferred electronically from one site to another.

In its current form, "near real-time" consulting relies on the use of shaky, television-like, low-quality, and low-frame-rate pictures for medical consultations. The technique has various drawbacks and limitations. First, the video feed's quality may hinder the accuracy of visual examinations and diagnoses, making it difficult for healthcare practitioners to obtain an accurate understanding of a patient's condition. Second, the low frame rate may cause lag and lower communication fluidity, which may hinder efficient doctor-patient interactions [9].

Teledentistry offers a transformative opportunity to expand the reach of oral healthcare, enhance its delivery methods, and reduce associated costs. Moreover, it holds the potential to ameliorate the oral health disparities that often persist between rural and urban areas. A particularly effective and efficient strategy to address the healthcare divide between these distinct populations could lie within the domain of teledentistry [5].

By leveraging technological advancements, teledentistry can effectively overcome geographical barriers, extending its services to individuals who previously had limited access to oral healthcare. This paradigm shift in healthcare delivery not only increases the inclusivity of oral health services but also optimizes the utilization of resources, ultimately leading to a more sustainable healthcare ecosystem [10].

Teledentistry in pediatric and preventive dentistry

As part of a child's treatment plan, a dental clinic or hospital may need to execute a compressive approach, including various specializations like operative, endodontic, orthodontic, or surgery. For the treatment of teenage dental issues, a model of teledentistry-assisted therapy should be developed. This model may be supplemented with video-based and live teleconsultation demos. It offers non-emergency at-home advice that may be provided to parents and carers via teleconsultation. The major purpose of this technique is to aid patients who are unable to acquire decisive care from a professional owing to infection risk, hospital overpopulation, and a shortage of functioning dental clinics. Teledentistry can help with pediatric patient behavior counseling, pediatric patient diagnosis, and monitoring in faraway locations that have limited availability of dental care, and pediatric patient promotion of oral health. By keeping a safe distance, this technique can be especially helpful in the pandemic crisis to reduce patient-to-patient contact. The usage of smartphones, webcams, intra-oral cameras, and Internet-connected dentistry apps minimizes the danger of exposure to dental personnel and practitioners [11].

Caries suppression is mostly achieved through early detection and prevention of this widespread disease. In many circumstances where direct clinical examinations are impractical, teledentistry has emerged as the method of choice. It has been proven in practice that non-invasive imaging-based remote diagnosis of pediatric dental issues provides a reliable foundation for an accurate understanding of dental problems. The quality of intraoral cameras plays a major role in the increasing success rate of teledentistry [12].

Children are more skilled at using digital technology than adults are, and they are more immersed in it. Dental practitioners can routinely connect with their patients using social networking technologies. Finally, the use of the Internet and mobile-based apps improves patient understanding and comprehension of oral hygiene promotion, provides an effective form of interaction for parents and the community in remote areas, and hides a dearth of qualified dental public health workers. They are easy to use since the applications can be accessed immediately from a smartphone, eliminating the need for an additional device, and feedback, as well as precise reminders and instructions for new behaviors or habits, may be sent following a real-time evaluation of the user [13].

Mobile gaming apps provide youngsters with a hands-on approach to managing their conduct and help facilitate face-to-face interaction by lowering the frequency of dentist visits. These applications may employ behavior coaching tactics such as "tell-show-do," "positive pre-visit counseling," "distraction," and "modeling" [14]. Previous research has demonstrated that video modeling may be therapeutic in the treatment of anxiety while simultaneously being instructive in the improvement of children's coping capacities in stressful situations. A peer model that deals with comparable dental operations that the child has undergone outperforms irrelevant models or user videos [10,15].

Benefits for patients

In emergency situations, patients have the valuable option to directly reach out to a dentist from virtually anywhere, thanks to teledentistry. This innovative approach empowers dentists to conduct a detailed evaluation of the issue before recommending any treatment or medication, resulting in significant time and cost savings. It also reduces the urgency for immediate visits to hospitals or dental clinics. Consequently, this approach minimizes the need for travel and leads to shorter waiting times at dental offices. Teledentistry offers these benefits while maintaining high-quality dental care standards. Additionally, teledentistry gives patients and their families the freedom to pick a dentist who fits their unique preferences and requirements. This makes it possible to consult a dental professional for a second opinion, even if they are far away from the patient [6,16].

Benefits for dentist

The use of teledentistry has several benefits for patients and dentists. Patients spend a lesser amount of time in chairs when they don't have to go to the clinic as frequently, which is an important benefit. Virtual consultations with dentists enable the option of examining more patients in a single day. Additionally, it may not always be essential to schedule follow-up appointments following treatment because dentists can keep in touch with their patients virtually to check on their progress and deliver advice as needed. Teledentistry also facilitates quick access to specialist opinions when required, with the patient's consent. Dentists can efficiently consult with specialists, even if they are geographically distant, ensuring that patients receive the most comprehensive care and expert advice when necessary [16,17].

Scope of teledentistry in India

Teledentistry applications include tele-triage, teleconsultation, telediagnosis, and telemonitoring. Tele-triage assists in prioritizing patients who require urgent care by remotely assessing pathology and providing those seeking dental care with safe access. Tele-diagnosis is the use of photos to evaluate oral disorders from a distance. Patients no longer need to travel as far for dental care, especially those in distant places who confront socioeconomic and geographic challenges. Telemonitoring, teleconsultation, and tele-triage lessen this need [13].

India has also opened up to telemedicine in order to address a number of problems with the healthcare delivery system, including poor clinical services and infrastructure, a shortage of qualified medical professionals, a near-complete lack of access to specialized care, the late detection of illnesses, and delays in treatment due to the longer travel times patients must take to reach urban healthcare facilities [18].

In India, we are no longer able to offer complete basic healthcare in remote places. Secondary and tertiary dental treatment is not always easily accessible in suburbs and cities. Incentives to encourage professionals to practice have proven ineffective, especially in suburban regions [19].

In contrast to the dire situation in healthcare, India's level of computer literacy is rapidly improving. Telemedicine is increasingly considered a new avatar by healthcare practitioners. In principle, building a first-rate telecommunications network throughout suburban and rural India would be far less difficult than stationing hundreds of dental experts there. We have realized that the future of the telecoms business is bright [19].

Limitations in teledentistry

Clinicians navigating the intricacies of teledentistry might face challenges in embracing the technology, as the complexity of the field may render them hesitant to acquire and apply new skills. Despite harsh legal consequences for breaches of patient privacy without permission, a lack of privacy in communicated information may be an impediment to winning patients' trust. It is recommended that all healthcare providers and telehealth portals follow the Health Insurance Portability and Accountability Act (HIPAA).

In terms of communication, although numerous surveys are being conducted to demonstrate the growing popularity of teledentistry, patient acceptance may be hampered by a lack of in-person contact, which may impede the implementation of a specified treatment plan [20]. In rural settings, the lack of amenities such as connectivity to the Internet connection, computers, cellphones, X-ray equipment, and other sophisticated armamentarium might be used to diagnose the tooth or lesion, provide the patient with it, or transfer the patient to a specialist at a different site [20].

In real-life situations, the implications of insufficient interproximal touch and inspection of the posterior-most teeth, the inability to conduct a tactile examination of the lesion/oral cavity, and the limitations of two-dimensional images can hinder the accuracy of a diagnosis. Clinicians may experience anxiety about the potential for incorrect diagnoses and treatments [20].

Future prospective

Even though there are still numerous ethical and legal difficulties that need to be resolved, teledentistry has the potential to bring about a lot of exciting improvements in the years to come. The following are the numerous actions that can be taken to implement teledentistry successfully: a nationwide teledentistry training initiative, or maybe its incorporation into the dental studies curriculum, and appropriate licensure procedures. Professionals should ensure that their systems and data are secure. Adjuncts like password protection, user access logs, and data encryption should be kept up with and routinely reviewed [21].

Discussion

By using teledentistry, dental care can be delivered in isolated and rural areas where there are few opportunities for professional consultation. A developing country like India, which has a varied topography, a substantial rural population, and an established healthcare delivery infrastructure combined with telecommunications technology, makes a perfect environment for the effective implementation of teledentistry [20-22].

Teledentistry should be viewed as an innovative method for delivering established dental services rather than a distinct specialty within the field. Its significance becomes particularly evident when considering its applicability in remote and underserved areas, whether they be rural or urban. The primary purpose of teledentistry lies in overcoming the barriers to accessing specialist dental consultations in regions where such services are scarce. By leveraging telecommunications technology, it offers an efficient and effective means of connecting patients with dental professionals without the need for in-person visits [5]. Teledentistry can be conducted through various platforms, including mobile phones, video conferencing, and social media. According to several studies, video conferencing emerged as the most favored method, followed by phone consultations. However, a subset of participants expressed a preference for traditional face-to-face appointments, potentially indicating a lack of awareness regarding technology utilization or the absence of necessary tools for teledentistry [23]. Kopycka-Kedzierawski et al. conducted a study assessing the feasibility of using intraoral cameras and telehealth for screening dental conditions, including early childhood caries (ECC), in preschool-aged children. Employing the Dr. Camscope intraoral camera within the Health-e-Access telehealth, they evaluated the diagnostic quality of dental images. The study involved 50 preschoolers aged 4 to 6, each providing a comprehensive set of dental images. The results revealed no statistically significant difference between the intraoral camera examination and the traditional visual examination. This suggests that intraoral cameras could serve as a viable and cost-effective alternative for screening caries, particularly ECC, in preschoolers attending childcare centers [12].

A study was conducted by Kopycka-Kedzierawski et al. on 12- to 60-month-old children enrolled in early head start inner-city childcare centers to determine the prevalence of caries using teledentistry. Using an intraoral camera, skilled telehealth assistants captured images of the primary teeth, which were then assessed by a pediatric dentist with calibration. Sixty-nine of the 162 infants who underwent screening had ECC, while 93 were caries-free. The findings of this study indicate that teledentistry presents a potentially effective way to check for ECC symptoms in high-risk preschoolers [24].

In a feasibility study conducted jointly by the Chin-Shan Group Health Center and National Taiwan University Hospital in 2000, a single resident, equipped with an intraoral camera, a digital radiographic system, and specialized software for image transfer, was dispatched to serve the 17,000 residents of Chin-Shan Township. This innovative project demonstrated the effectiveness of teledentistry in providing dental healthcare to people living in remote areas. It also highlighted how useful it is to use this novel method to provide remote specialist consultations [25]. The University of Rochester's Eastman Department of Dentistry initiated a notable teledentistry program in 2006. Aiming to provide additional dental care services to a less fortunate population of young children, this creative initiative was launched throughout six inner city primary schools and seven child care centers. The outcome of the research was both informative and alarming since it exposed an important oral health problem. Nearly 40% of children between the ages of 12 and 48 months had active dental caries, according to the teledentistry screening procedure, showing a significant incidence of tooth decay in this age range [26]. The difficulty these young children in underprivileged urban areas face when maintaining their oral health was brought home by this finding, which highlighted the critical need for early dental care and preventative measures. The initiative highlighted the significance of addressing oral health inequalities among young children to maintain their general well-being, in addition to showing the efficiency of teledentistry in reaching such areas [26].

El Tantawi et al. conducted a study that offers insights into the adoption of teledentistry in 19 countries spanning various regions. The results reveal that a majority of these countries lack legal frameworks, policies, or regulatory bodies overseeing telehealth [26]. Even in instances where telehealth policies exist, many do not specifically address teledentistry. Frequently, teledentistry initiatives are informally implemented at the local level as projects [27].

In order to provide low-cost dental care, general dentists and dental hygienists may work in both the primary and secondary sectors of the healthcare delivery system. As part of their internship training program, recently graduated dentists may be assigned to these facilities, where they will learn about case diagnosis and treatment planning. Because dental schools hold a variety of experts under one roof, they are potential centers for teledentistry consultation. Every new endeavor comes with some drawbacks, such as the privacy of medical and dental information. Before teledentistry can fully develop and offer patients appropriate care, worries regarding the transmission of medical histories and records, security issues with electronic data saved on computers, and the mushrooming "cyber dentist" need to be addressed [4,28].

## Conclusions

The combination of innovative medical tools and cutting-edge technology in the present landscape of pediatric dentistry has revolutionized the area of teledentistry, changing it into an exceptionally convenient and successful technique for engaging and serving a wide range of young patients. Teledentistry, in its modern version, now includes teleconsultation support, which is available at any time and from any location via Internet-based platforms. This evolution goes far beyond the traditional in-person dental treatment approach, bringing various benefits that are particularly crucial in the current healthcare context. One prominent asset of teledentistry is its ability to raise public awareness of various oral health conditions and express essential data, particularly among the pediatric population. Teledentistry, by utilizing Internet-based media platforms, shows itself to be a powerful instrument for efficiently addressing a broad and targeted public, particularly during critical situations such as emergencies, where rapid and extensive communication is essential. In the field of pediatric dentistry, this implies that parents and carers can get timely advice and information, promoting improved oral health practices for children and ensuring that critical insights are quickly available to address emerging dental difficulties in the younger population.
